# Worsening Hypothyroidism in the Setting of New-Onset Nephrotic Syndrome

**DOI:** 10.7759/cureus.43345

**Published:** 2023-08-11

**Authors:** Qi Wang, Divya Ravi, Zhiting Tang, Nikita Nand, Ala Dahhan

**Affiliations:** 1 Internal Medicine, Rochester Regional Health, Rochester, USA; 2 Cardiology, Rochester Regional Health, Rochester, USA; 3 Nephrology, Rochester Regional Health, Rochester, USA

**Keywords:** primary membranous nephropathy, levothyroxine, proteinuria, nephrotic syndrome, hypothyroidism

## Abstract

We present a case of a 54-year-old female with well-controlled hypothyroidism who experienced worsening symptoms due to nephrotic syndrome. The patient presented with fatigue, progressive shortness of breath on exertion, and anasarca for one month. Laboratory results revealed significantly elevated thyroid-stimulating hormone levels and nephrotic range proteinuria. A kidney biopsy showed stage I membranous nephropathy with positive phospholipase A2 receptor (PLA2R) findings. Her symptoms gradually improved after receiving a higher dose of levothyroxine, along with diuretics and lisinopril initiation. She continued to be closely monitored by both endocrinology and nephrology outpatient services. This case report highlights the importance of closely monitoring hypothyroidism treatment when significant proteinuria is present.

## Introduction

The coexistence of hypothyroidism and nephrotic syndrome in a patient can lead to complex clinical challenges, where the treatment of one condition may impact the other. Hypothyroidism, characterized by insufficient thyroid hormone production, and nephrotic syndrome, characterized by significant proteinuria and edema, represent two distinct medical conditions with distinct management strategies. It has been reported that urine loss of protein can cause hypothyroidism [1], and their interactions can complicate the clinical presentation and management of affected individuals [2-4]. In this report, we discuss a case of a 54-year-old female with well-controlled hypothyroidism who experienced worsening symptoms due to nephrotic syndrome, shedding light on the importance of closely monitoring hypothyroidism treatment when significant proteinuria is present.

## Case presentation

A 54-year-old female with a history of papillary thyroid carcinoma s/p total thyroidectomy and iodine treatment and postoperative hypothyroidism for 10 years presented with fatigue, shortness of breath on exertion, anasarca, and weight gain for one month. She also had constipation and cold intolerance for the same time frame. She noted puffy eyes and foamy urine for about one week. The patient had postoperative hypothyroidism for 10 years and was well controlled, with thyroid-stimulating hormone (TSH) ranging between 0.45 and 2 during the past two years. She was maintained at 150 mcg and her follow-up thyroid antibodies as well as imaging had been normal. However, her TSH was found to be 48.14 two months prior to her admission, for which she saw endocrinology. Her levothyroxine was increased to 175 mcg. The patient reported she never missed a dose and has always waited one hour before taking her meal. Despite increasing the dose and being compliant, she started to get symptomatic and TSH kept trending up.

On presentation, she was noted to be in sinus bradycardia but otherwise vitally stable. Anasarca with pitting edema was noted on her physical exam. Labs were significant for elevated TSH at 48.14 with low-normal free T4 at 0.9, urinalysis showed significant proteinuria >= 1000, moderate blood, and elevated red blood cell (RBC) at 21-40. The urine protein creatinine ratio was at 4731 mg/g. Her albumin was mildly decreased to 3.2. Cholesterol and triglycerides were also elevated (Table 1). Transthoracic echocardiogram (TTE) was unremarkable.

**Table 1:** Basic laboratory findings WBC: white blood cells count; TSH: thyroid-stimulating hormone; HDL: high-density lipoprotein; LDL: low-density lipoprotein; BNP: brain natriuretic peptide; NT-proBNP: N-terminal pro-b-type natriuretic peptide.

Lab test	Reported value	Normal value	Units
Hemoglobin	15.5	11.5-16.0	g/dl
WBC	5700	4000-10000	cells/ul
TSH	70.47	0.55-4.78	uIU/ml
Creatinine	1.1	0.5-1.1	mg/dl
24-hour urine protein	5854	0-150	mg/24 hours
Total cholesterol	320	0-199	mg/dl
HDL	77	40-60	mg/dl
LDL	223	0-99	mg/dl
Antithyroglobulin antibody	<15	0.0-59.9	U/ml
Thyroglobulin	<0.2	<55.0	ng/ml
Random cortisol	9.7	3.0-23.0	ug/dl
Troponin I, high sensitive, 0 hours	5	0-50	pg/ml
Troponin I, high sensitive, 1 hour	5	0-51	pg/ml
Troponin I, high sensitive, delta 0-1 hour	0	0-9	pg/ml
BNP	36	0-100	pg/ml
NT-proBNP	119	0-124	pg/ml

**Figure 1:**
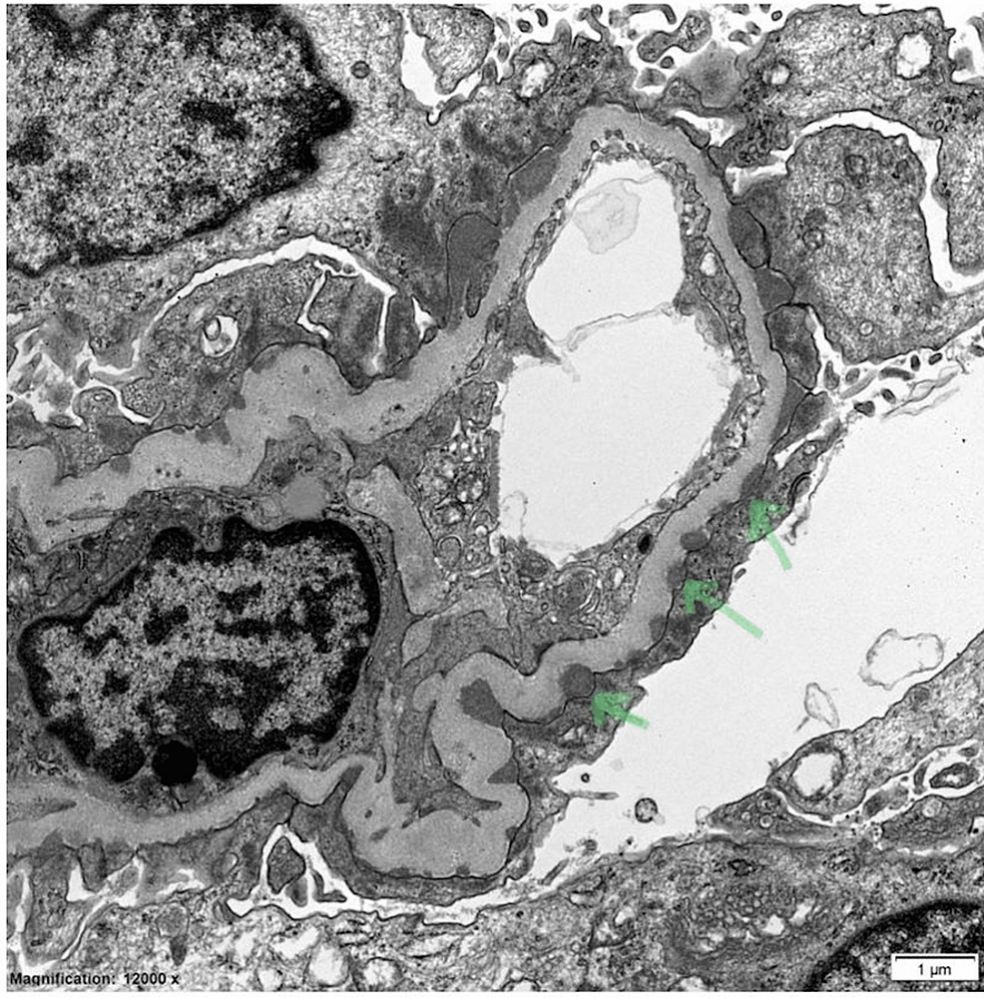
Stage I membranous nephropathy Arrows: Diffuse, small, sub-epithelial, electron-dense, immune-type deposits.

**Figure 2:**
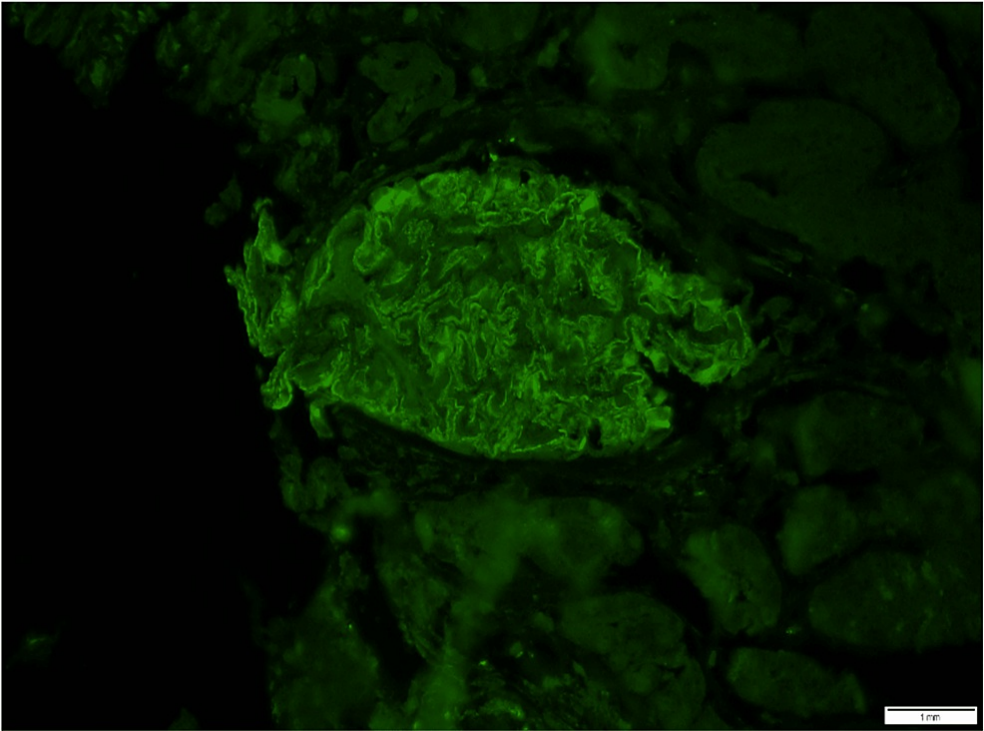
Positive phospholipase A2 receptor (PLA2R) staining observed on biopsy Positive and finely granular PLA2R staining.

Extensive serological and infectious laboratory tests yielded negative results for our patient with new-onset nephrotic syndrome (Table 2). She did not receive any non-steroidal anti-inflammatory drugs (NSAIDs) or antibiotics prior to her presentation. Kidney biopsy revealed stage I membranous nephropathy (Figure 1), with positive phospholipase A2 receptor (PLA2R) staining observed on biopsy (Figure 2), and negative serum PLA2R levels. Previous cancer screening, including colonoscopy, mammogram, and pap smear, did not reveal any signs of malignancy. Thyroid antibodies and ultrasound were regularly monitored every two years, and there were no indications of relapse.

**Table 2:** Extensive laboratory findings

Lab test	Reported value	Normal value	Units
Total protein	4.8	5.7-8.2	g/dl
Albumin	2	3.4-5.2	g/dl
Total globulin	2.9	1.7-4.3	g/dl
Alpha 1	0.2	0.1-0.4	g/dl
Alpha 2	0.9	0.4-1.0	g/dl
Beta	1	0.8-1.4	g/dl
Gamma	0.8	0.4-1.5	g/dl
Paraprotein	Not detected	Not detected	g/dl
C-reactive protein	103	0-10	mg/L
Complement C3	170	90-180	mg/dl
Complement C4	37	18-45	mg/dl
Antinuclear antibody screen	Negative	Negative	AI
Cryoglobulin	Negative	Negative	AI
Anti-ribonucleoprotein antibody	Negative	Negative	AI
Anti-Smith antibody	Negative	Negative	AI
Hepatitis B surface antigen	Negative	Negative	AI
Hepatitis C antibody	Negative	Negative	AI
Kappa free light chains	2.76	0.3300-1.94	mg/dl
Lambda free light chains	2.69	0.5700-2.63	mg/dl
Kappa/lambda free ratio	1.03	0.2600-1.65	

Based on the above findings, we concluded that this is a case of inactive primary membranous nephropathy. As a result, the decision was made to proceed with supportive management, without administering steroids or non-steroidal immunosuppressive agents. Endocrinology and nephrology departments closely monitored her progress, and since her discharge, she has been maintained on a daily regimen of levothyroxine (300 mcg), lisinopril (5 mg), and furosemide (40 mg). Her most recent TSH level improved to 17, and her symptoms have gradually improved as well.

## Discussion

Thyroid hormones are predominantly bound to plasma proteins such as thyroid hormone-binding globulin (TBG), transthyretin (prealbumin), and albumin, as they are hydrophobic in nature [5]. In patients with nephrotic syndrome, these proteins tend to be lost in the urine, and the degree of hypothyroidism appears to be associated with the severity of proteinuria, as indicated by a retrospective cohort study conducted by Kwong et al. in 2021 [6].

Furthermore, it has been observed that thyroid hormones can also be excreted in the urine. Fonseca et al. [1] conducted a study in 1991, measuring urine thyroxine (T4) levels in 10 patients with nephrotic syndrome. T4 was detected in the urine of five out of the 10 patients. Additionally, both serum free T4 and free triiodothyronine (T3) levels were found to be inversely correlated with urinary T4 levels. However, there was no significant difference observed in serum thyrotropin levels.

Currently, there are no specific guidelines regarding the necessary increase in levothyroxine dosage for patients with thyroid dysfunction and nephrotic syndrome. However, a study by Karethimmaiah et al. (2016) [2] observed nine patients who had previously been diagnosed with primary hypothyroidism and later developed nephrotic syndrome. The study found that these patients required a 17.6% increase in their replacement dose of levothyroxine to achieve improvements in symptoms and laboratory results. In reality, other different methods were also tried. As the case reported by Iqbal et al. (2022) [3], an extra-oral liothyronine 5 mcg daily was added in addition to the existing 100 mcg of levothyroxine while initiating a gel capsule of levothyroxine at the same dose to achieve better absorption. Chandurkar et al. (2008) [4] reported a case in which the required dose of levothyroxine increased from 150 mcg to 200 mcg daily, which was a 33.3% increase. In our patient's case, a 71.4% increase in levothyroxine dosage was necessary to achieve clinical improvement. This higher dosage may be attributed to the persistent proteinuria, albeit with gradual improvement, as well as factors such as obesity and anasarca, which could decrease the absorption of levothyroxine.

The difference in approaches to the management of thyroid dysfunction noted in previous cases may be due to the different pathologic causes of the nephrotic syndrome as cases where prompt improvement of proteinuria may not require large dosing changes of the levothyroxine. Usually, those patients were able to go back to their previous dose or the dose close to their previous dose after six to 12 months once the resolution of proteinuria was achieved [3,4,7].

In summary, this case involves a newly diagnosed inactive primary membranous nephropathy and a subsequent decline in previously well-controlled hypothyroidism. The patient received supportive management and was prescribed a higher dose of levothyroxine, resulting in gradual improvements in both laboratory values and clinical symptoms.

## Conclusions

The new onset of nephrotic syndrome can cause thyroid dysfunction with previously well-controlled hypothyroidism, and the degree of hypothyroidism is related to the severity of proteinuria. Most patients with hypothyroidism in the setting of nephrotic syndrome require adjustment of hypothyroidism treatment, although there is no specific guideline in terms of dose adjusting. As healthcare providers, it is crucial to be aware of the association between nephrotic syndrome and hypothyroidism, as timely investigation and treatment for both conditions are necessary. It is also reasonable to consider assessing thyroid function in patients with newly diagnosed nephrotic syndrome.
